# Pregnant Women’s Risk Perception of the Teratogenic Effects of Alcohol Consumption in Pregnancy

**DOI:** 10.3390/jcm8060907

**Published:** 2019-06-25

**Authors:** Isabel Corrales-Gutierrez, Ramon Mendoza, Diego Gomez-Baya, Fatima Leon-Larios

**Affiliations:** 1 Foetal Medicine Unit, University Hospital Virgen Macarena, 41009 Seville, Spain; icorrales@us.es; 2 Department of Surgery, University of Seville, 41009 Seville, Spain; 3 Department of Social, Developmental and Educational Psychology, University of Huelva, 21007 Huelva, Spain; ramon@dpsi.uhu.es; 4 Research Group on Health Promotion and Development of Lifestyle across the Life Span, University of Huelva, 21007 Huelva, Spain; 5 Center for Research in Contemporary Thought and Innovation for Social Development (COIDESO), University of Huelva, 21007 Huelva, Spain; 6 Nursing Department, Faculty of Nursing, Physiotherapy and Podiatry, University of Seville, 41009 Seville, Spain; fatimaleon@us.es

**Keywords:** risk perception, alcohol consumption, pregnancy, FASD, prevention, Spain

## Abstract

There is ample evidence of the teratogenic effects of prenatal alcohol exposure, with long-term consequences throughout the entire life cycle. Nevertheless, research on risk perception of alcohol consumption among pregnant women is scarce. In order to analyze risk perception of alcohol consumption during pregnancy, a cross-sectional study was conducted with a random sample of 426 pregnant women (in their 20th week of gestation) receiving care at the outpatient clinics of a public university hospital in the southern European city of Seville (Spain). Data were collected through structured face-to-face interviews conducted by trained health professionals using a customized questionnaire. Data analysis included structural equation modeling. Only 48.1% of the sample indicated that the sequelae from alcohol consumption during pregnancy were life-long. The structural equation model showed that a lower risk perception about beer and wine consumption, and a lower educational level, were related to more frequent alcohol consumption. Younger participants showed lower risk perception concerning beer consumption. Higher levels of education were related to a greater risk perception of beer. Healthcare institutions should articulate programs that facilitate health advice regarding alcohol consumption during pregnancy, particularly when providing care for women with low educational levels.

## 1. Introduction

The scientific community’s perception of alcohol as a teratogenic agent arose with the publication of Lemoine’s pioneering study [1], which sparked interest in the harmful effects of intrauterine exposure to alcohol. However, it was not until 1973 that the diagnostic term “Fetal Alcohol Syndrome” (FAS) was coined to describe the pattern of malformations seen in children of mothers who chronically consumed alcohol during pregnancy [2]. The term refers to a specific set of malformations or defects that may occur in the fetus due to a mother’s alcohol consumption during pregnancy. Today, the term “Fetal Alcohol Spectrum Disorders” (FASD) is an umbrella term to describe the wide range of neurocognitive disorders, congenital malformations, and other physical and behavioral problems resulting from prenatal exposure to alcohol [3,4,5]. Alcohol consumption during pregnancy has become the leading non-hereditary preventable cause of intellectual impairment [6].

There is ample evidence of the teratogenic effects of prenatal alcohol exposure, with long-term consequences throughout the entire life cycle [7,8,9,10,11]. Regarding the consumption of low doses of alcohol during pregnancy, although there are animal studies and descriptive studies on humans showing that it may be teratogenic [12,13], consensus on the matter has not yet been achieved. However, in application of the principle of precaution, abstaining from any type of alcohol consumption during pregnancy is the only safe option [14,15,16,17].

Alcohol consumption during pregnancy shows great epidemiological variation worldwide. A systematic review and meta-analysis estimated the global prevalence of alcohol consumption during pregnancy to be 9.8%, but there are countries where the estimated prevalence exceeds 45%, as is the case of Ireland with 60.4%, Belarus with 46.6%, and Denmark with 45.8% [18].

Notwithstanding, some studies suggest that those studies based on self-report measures underestimate the prevalence of alcohol consumption in pregnancy, when comparing data provided by women with those obtained analyzing biomarkers, which could provide a more accurate evaluation of alcohol consumption patterns throughout pregnancy [19,20].

Numerous studies have been able to show an inverse relationship between the risk perception of the use of a particular harmful substance or drug (cannabis, alcohol, or tobacco) and the levels of use of that substance or drug in a social group [21,22,23,24]. However, concerning alcohol specifically and its consumption during pregnancy, there are very few studies exploring pregnant women’s risk perception and beliefs about the possible harmful effects of alcohol consumption during pregnancy [25,26]. In turn, risk perception and beliefs regarding the effects of alcohol on pregnancy vary as a function of the information provided by the healthcare personnel [27]. Nevertheless, some studies suggest that health advice regarding alcohol consumption during pregnancy is not a very widespread practice among those practitioners involved in standardized pregnancy care, or that there are inconsistencies in the information about alcohol use during pregnancy and in the advice provided [28,29]. Specifically, in a study carried out in a southern European city with a random sample of pregnant women, 43% of the interviewees stated that they had not received any health advice on this matter. Only 43.5% of the sample remembered having received the correct message (not to consume any alcohol at all during pregnancy) from their midwife, 25% from their obstetrician, and 20.3% from their GP. Women with a low educational level were those who stated having received health advice on the issue to the least extent [29].

Research on risk perception of alcohol consumption among pregnant women is scarce, particularly in the Mediterranean countries of Europe, which have traditionally been characterized by a high level of alcohol consumption (among women as well). Specifically, almost no research has been done on whether this perception varies as a function of the type of drink (beer, wine, or liquor) in question [30]. 

There is also very little research available on whether a pregnant woman’s perceived risk of alcohol consumption during pregnancy varies as a function of her obstetric history, although some studies suggest that the perceived risk of alcohol consumption in pregnancy is lower in those women who have previously given birth to a healthy child [26].

Similarly, few studies have looked at whether a pregnant woman’s risk perception varies as a function of her sociodemographic characteristics. A directly proportional relationship has been shown between education level and risk perception of alcohol consumption during pregnancy, with those pregnant women with the higher levels of education showing greater risk perception [31].

Therefore, our study, conducted on a sample of pregnant women receiving care in the outpatient clinics of a public hospital in a southern European city, was aimed at determining pregnant women’s risk perception of alcohol consumption during pregnancy, and whether said perception varies as a function of their education level, or as a function of obstetric factors, such as the number of previous pregnancies. Similarly, the study also looked at whether their beliefs about the potential damage resulting from consuming alcohol during pregnancy vary with respect to the type of alcoholic beverage being considered (fermented vs distilled). Lastly, we examined the relationship that exists between a pregnant woman’s risk perception of alcohol consumption during pregnancy and her own level of alcohol consumption during her pregnancy, taking into account their sociodemographic and obstetric characteristics. 

Our initial hypotheses were, first, that the higher the pregnant woman’s education level, the higher her risk perception of consuming alcoholic beverages during pregnancy will be. Second, that multiparous women will be, in general, less conscious of this risk than nulliparous women. Third, that this risk perception varies as a function of the type of alcoholic beverage in question, being higher for distilled beverages than for fermented beverages. Lastly, it was hypothesized that women’s risk perception of the potentially harmful effects of alcohol on the pregnancy modulates their own consumption during their pregnancy, so we expected to find an inverse relationship between perceived risk and alcohol consumption during pregnancy.

## 2. Participants and Method

### 2.1. Study Design 

A cross-sectional study was conducted on a representative sample of pregnant women attending the 20th-week scan at the outpatient clinics of a public university hospital in the city of Seville (Spain).

### 2.2. Data Collection and Participants

The participants included 426 pregnant women in their 20th week of pregnancy (the desired sample size was 400). The data were collected through structured face-to-face interviews conducted by trained health professionals, using an ad hoc anonymous questionnaire. 

The sample was obtained by randomly selecting one out of every two pregnant women who were coming in for their 20th-week pregnancy ultrasound at the outpatient clinics of the university hospital during a five-month period in 2016. To be eligible for inclusion, women should be aged 16 years and over, be able to read and speak Spanish fluently, and complete an informed consent form.

The population in the geographical area served by this public hospital is quite similar to the average Andalusian population, in terms of the percentage of women in reproductive age (43.1% and 43.6%, respectively), work activity rates among women (49.2% vs. 49.0%), unemployment rates (20.8% vs. 18.7%), and education level (e.g., the percentage of women with low level of studies was 23.4% in the study area and 24.0% in Andalusia, according to the 2011 population census). Thus, educational level and employment situation of the sample is quite similar among women in this specific age period in the region [29]. 

### 2.3. Ethics

Before starting the survey, the study protocol and the questionnaire were approved by the Clinical Research Ethics Committee of University Hospital Virgen Macarena (Research code: ICG15/ Internal code: 0254N-15). 

Prior to the interview, the participants were all provided with an informed consent form, which included information about the purpose of the study and the voluntary nature of their participation, as well as the guarantees for privacy and confidentiality and how their information would be handled anonymously. The participants gave their informed consent by signing and returning this form. The Helsinki declaration of 1975 and its subsequent amendments were respected.

### 2.4. Questionnaire

In order to gather the data, a customized questionnaire was designed by the research team, taking into account their professional experience as health providers as well as their personal experience in previous research on lifestyle and risk perception. A few questions (those regarding alcohol consumption patterns) were selected from the Alcohol Use Disorders Identification Test (AUDIT) [32]. Most of the questions of the questionnaire contained fixed choice response options, but included a final option for “other response”, where the interviewer could note down all the spontaneous answers given by pregnant women that apparently did not fit with the established categories. The transcription of these notes was later studied by the research team, in order to decide whether they could be adapted to any of the foreseen answer categories or they should be considered as a different category. In addition, some questions of the questionnaire were open-ended and participants’ responses were recorded via note-taking. For these questions, the categories were created after data collection, based on a thematic analysis of participants’ responses, and the response of each interviewee was then coded according to this categorization. The questionnaire was piloted in order to verify that all questions were understandable and to consider whether new response categories in specific questions should be added.

(a) Socio-demographic variables: age, educational level (sorted into three groups: (1) low level of studies; i.e., primary school or lower; (2) medium level of studies; i.e., compulsory secondary school, post-compulsory secondary school, and vocational training; (3) university studies), employment status (sorted into four groups: full-time work, part-time work, unemployed, and other employment status) and relationship status.
(b) Obstetric variables: number of pregnancies, including the current one, and pregnancy planning.
  General belief about whether alcohol consumption during pregnancy may be harmful to the pregnant woman herself or to her baby. The principal response options were “Yes, to the baby”, “Yes, to me”, “Yes, to both”, “No” and “I don’t know”. These response categories were fixed after piloting the questionnaire. In addition, an open-ended choice of “Other response” was foreseen. The content of this response, when filled, was analyzed after the data collection as described above.
(c) Type of risk resulting from alcohol consumption during pregnancy. If the participant gave an affirmative response to the previous question, she was then asked to name the risks that she thought resulted from alcohol consumption during pregnancy. This was an open-ended question and the response was written down literally. After data collection, following the process described above, the following main response categories were established: malformation, miscarriage or abortion risk; premature delivery risk; withdrawal syndrome risk; problems in the baby’s development risk.
(d) Belief about how long the problems resulting from alcohol consumption during pregnancy can last, with the following response categories: “Only during the pregnancy”, “Only at birth”, “During the first years of life”, “For many years”, “Throughout the baby’s life”, and “I don’t know”. These response categories were fixed after the pilot study. Additionally, a final open-ended category could collect a different response of the interviewee. When this was the case, the categorization of this response was decided by the research team after data collection, as described above.
(e) Belief about the risk inherent in drinking alcoholic beverages in general during pregnancy as a function of the amount consumed and the frequency of consumption, with the following response options: “Any amount during pregnancy is harmful”, “Consuming alcohol less than once a month is not harmful”, “Consuming alcohol less than once a week is not harmful”, “Drinking a small amount every day is not harmful”, “Drinking as much and as often a person wants to is not harmful”. These categories were decided by the research team after studying the responses obtained to this question during the pilot. In the final questionnaire, a final open-ended category (“Other response”) allowed the team to write down literally any other response given by the interviewee, the categorization of which was decided by the research team prior to data recording.
(f) Belief about the risk inherent in alcohol consumption during the pregnancy as a function of the type of beverage (beer, wine, or distilled beverages), the amount consumed and the frequency of consumption, with the same five response options as the preceding question.
(g) Frequency of alcohol consumption during pregnancy. Using questions of the AUDIT scale [30], patterns of self-reported alcohol consumption during pregnancy were recorded.

### 2.5. Data Analysis

First, a univariate analysis of all the study variables was performed, evaluating the percent distribution of the various response categories. Second, differences in beliefs and in the self-reported frequency of alcohol consumption were analyzed as a function of demographic variables, applying χ^2^ tests and linear-by-linear tests to the corresponding contingency tables. Third, applying these same tests, the relationship between the participants’ beliefs about the risk of alcohol consumption and their own consumption of alcohol was examined. These analyses were all performed using SPSS 21.0. Fourth, a path analysis was tested on the basis of the associations between variables observed in the previous analyses. In order to test the overall data fit of the model, χ^2^, CFI, and RMSEA were examined. The Lagrange multipliers test was conducted to improve the global fit of the model based on the modifications that were introduced. R^2^ was calculated to estimate how much of the variance of the study variables was explained by the model. This model was developed and tested using the EQS 6.3 statistical program.

## 3. Results

### 3.1. Descriptive Characteristics of the Sample

Out of a total of 1664 pregnant women who received care in these outpatient clinics throughout the data collection period, half of them (832) were asked to participate in the study and 426 (51.2%) agreed to participate. More than 90% of the participants (92.2%) had been born in Spain. Their mean age was 31.9 years (SD = 5.3).

Table 1 shows the characteristics of the sample by age, educational level, employment status, relationship status, and number of pregnancies, including the current one. Most participants were aged 31 years and over (64.3%) and almost all (98.1%) reported being in a relationship at the time of data collection. With respect to the level of education, a significant percentage of the women (45.5%) had medium level of studies, while more than a third reported university studies. Women who were employed full time were 39% of the sample, while 28% reported being unemployed. Finally, this was the first pregnancy for around 40% of the participants.

### 3.2. Self-Reported Frequency of Alcohol Consumption during Pregnancy

Three quarters of the women surveyed (75.4%) reported that they had not consumed any alcoholic beverages “during the last 4 or 5 months”, a period which presumably coincides with the amount of time that they have been pregnant, since they were, on average, in their 20th week of pregnancy. Of those who did report having consumed alcohol during said period, the most frequent response was “once a month or less” (14.6%). Only 1.2% reported drinking alcohol “2 to 3 times a week” (Table 1).

### 3.3. Beliefs about the Risks of Alcohol Consumption during Pregnancy

Based on participants’ responses, some categories were generated to analyze beliefs about risks. Almost half (48%) of the pregnant women indicated that consuming alcohol during pregnancy implied some risk for the baby. A significant percentage (42.6%) said that it involved a risk to both the baby and themselves, and 6.6% responded that it did not involve a risk when consumed in moderate quantities, or only when abused (Table 2).

When those women who did believe that it involved a risk were asked to specify, in their opinion, the type of risk involved, 27.1% responded that they did not know and failed to specify any risk. In turn, the problems mentioned most frequently by those who did specify types of risks were: “congenital malformations, spontaneous or induced abortion” (72.9%) and the generic term of “developmental problems for the baby” (24.6%). Only 2.5% of the women mentioned the risk of premature birth.

When the women who did indicate specific risk types were then asked how long they thought that the harmful effects derived from consuming alcohol during pregnancy could last, 48.1% indicated that the harmful effects were life-long, while 15.5% mentioned relatively brief time periods (the duration of the pregnancy, the birth, or the first years of life). Approximately one quarter of the women (27.5%) said they did not know how long the sequelae from alcohol consumption during pregnancy could last. 

When questioned about the risk perception of alcohol consumption during pregnancy in terms of the amount of alcohol consumed and the frequency of consumption, 69.7% of the women responded that any alcohol consumption was harmful. Conversely, only 0.4% of the participants said that drinking as much and as often as a person wants to drink is not harmful. In turn, 3.9% felt that “a small amount every day is not harmful”. 

When asked specifically about the risks involved in consuming beer or wine during pregnancy, the percentage of women who said that “any amount consumed during the pregnancy is harmful” was about half of those who gave the same response when asked about alcohol consumption in general (31.5% for beer and 38.4% for wine). On the other hand, the percentage who felt that “drinking a small amount every day is not harmful”, was 14.8% for beer and 5.9% for wine. Yet when asked the same question specifically about distilled beverages, the percentage who responded that “any amount consumed during pregnancy is harmful” jumped to 88%, and the percentage who said that “drinking a small amount every day is not harmful” dropped down to 1.9%. 

### 3.4. Sociodemographic Differences in Beliefs about the Risks of Alcohol Consumption during Pregnancy

**Figure 1:**
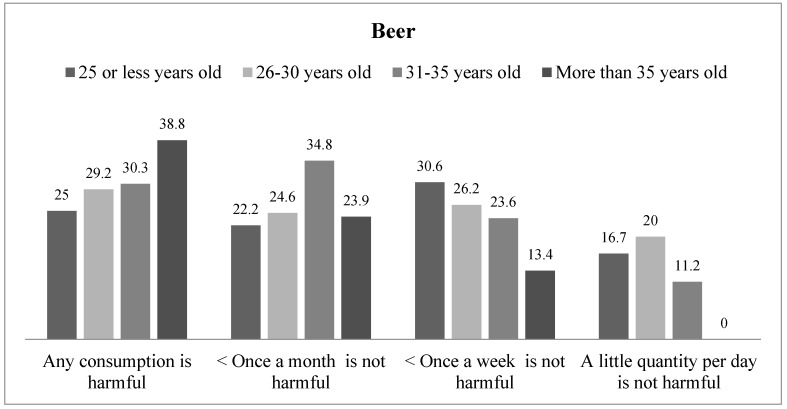
Risk perception of beer consumption in pregnancy by age.

There were age differences in risk perception of alcohol consumption during pregnancy (Figure 1). Younger participants reported less risk perception, so that 30.6% of women aged 25 or less stated that drinking beer once a week is not harmful, compared to 13.4% of those women aged 35 or older. Linear-by-linear test (1, N = 257) = 4.82, *p* = 0.028, γ = 0.15. One in four women aged 25 or younger reported that any beer consumption is harmful, while this response was more frequent among women aged 35 or older (38.8%). 

In turn, 64% of primiparous women reported some specific risk, in comparison to 52.7% of multiparous women. These differences were close to, but did not reach, statistical significance. 

**Figure 2:**
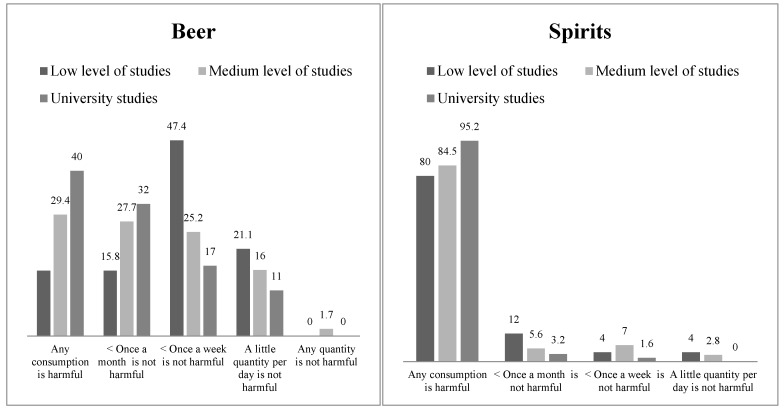
Beliefs concerning risks of drinking beer or spirits by educational level.

There were also differences as a function of educational level. Those participants with a higher level of studies showed higher perceived risk of harm resulting from drinking beer or distilled beverages in pregnancy. Figure 2 shows the risk perception of drinking beer and spirits as a function of the participant’s educational level. Concerning the risk perception of beer consumption, 47.4% of participants with low educational levels reported that drinking beer once a week while pregnant is not harmful, compared to 17% of the women with university studies, χ^2^(8, N = 257) = 22.51, *p* = 0.004, γ = 0.30. Moreover, only 15.8% of women with low educational levels reported that any beer consumption during pregnancy is harmful, in contrast with 40% of women with university studies. Regarding the risk perception of drinking distilled beverages, up to 95.2% of women with university studies said that any consumption was harmful, while 80% of women with low educational levels indicated the same thing, linear-by-linear test (1, N = 158) = 4.60, *p* = 0.032, γ = 0.45. Of the participants with low educational levels, 12% reported that consuming spirits once a month or less is not harmful, compared to 3.2% of participants with university studies. No significant differences by educational level were observed regarding risk perception of drinking wine, χ^2^(8, N = 219) = 6.63, *p* = 0.577. 

Furthermore, differences as a function of educational level were also found when specifying the type of risk resulting from the consumption of alcohol during pregnancy. Around 69% of participants with university studies were able to report some risks, while it was less frequent among participants with lower educational levels (52.9%), χ^2^(6, N = 402) = 17.81, *p* = 0.007, γ = 0.21.

**Figure 3:**
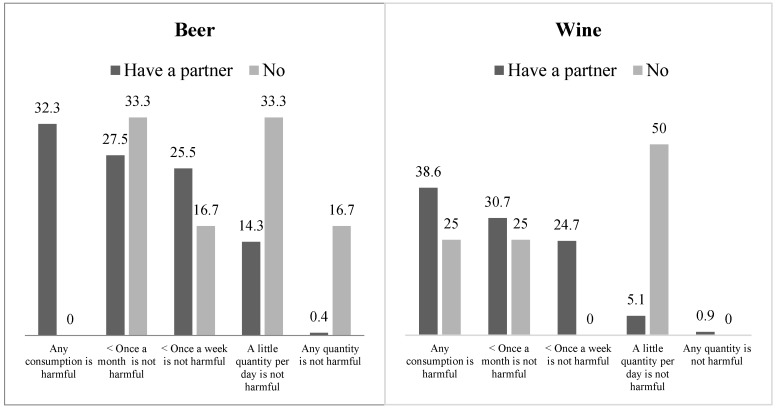
Risk perception of drinking beer or wine during pregnancy as a function of relationship status.

Additionally, a lower risk perception regarding beer and wine consumption was observed among participants who were not in a relationship at the time of the study (around 2% of the sample). Figure 3 presents the percentages for the beliefs concerning risks of drinking beer and wine as a function of relationship status. Half of women who were not in a relationship indicated that a small amount of wine per day was not harmful, compared to 5.1% of women who were in a relationship, χ^2^(4, N = 219) = 14.58, *p* = 0.006, γ = −0.41. In turn, 38.6% of participants in a relationship reported that any wine consumption is harmful, a higher percentage than reported by those who were not in a relationship (25%). Regarding beliefs about beer consumption, a third of the women who were not in a relationship indicated that daily consumption of a small amount of beer was not harmful, compared to 14.3% of the women in a relationship, χ^2^(4, N = 257) = 23.55, *p* < 0.001, γ = −0.61. None of the single women reported that any consumption of beer is harmful, in contrast with 32.3% of women in a relationship. 

### 3.5. Differences in Self-Reported Alcohol Consumption as a Function of Educational Level

**Figure 4:**
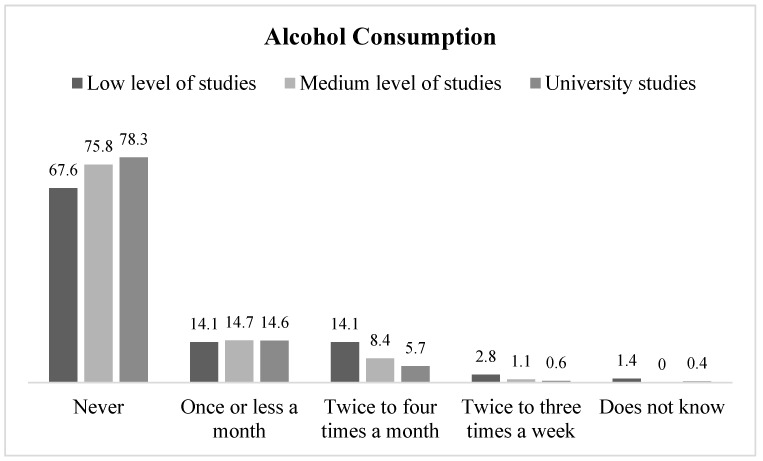
Frequency of alcohol consumption during pregnancy by educational level.

Participants with a lower educational level self-reported a greater degree of alcohol consumption, linear-by-linear test (1, N = 418) = 4.84, *p* = 0.028, γ = −0.16. Around 78% of women with university studies never drank alcohol, compared to 67.6% of women with low educational levels (Figure 4). Moreover, 14.1% of the women with low educational levels reported that they drank alcohol twice to four times a month, a frequency which was reported by 5.7% of women with the highest educational level. No significant differences were observed concerning other demographic variables.

### 3.6. Differences in the Frequency of Alcohol Consumption during Pregnancy as a Function of Risk Perception

**Figure 5:**
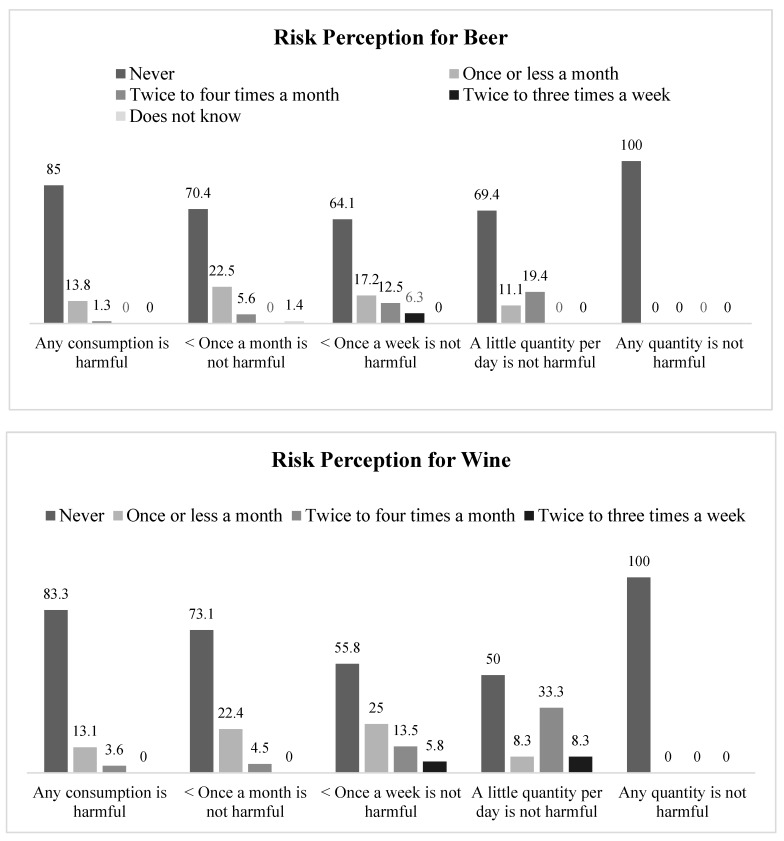
Frequency of alcohol consumption during pregnancy by risk perception of drinking beer and wine.

Figure 5 presents the results of the frequency of alcohol consumption during pregnancy as a function of risk perception of drinking beer and wine during pregnancy. A higher risk perception of these fermented drinks was associated with a lower alcohol consumption during pregnancy. 

Thus, regarding beliefs about the risk of beer consumption, 85% participants who indicated that any beer consumption is harmful reported no alcohol consumption, in contrast with 65.1% of participants who indicated that drinking one beer a week or less is not harmful, χ^2^(16, N = 252) = 32.25, *p* = 0.009, γ = −0.29. Moreover, among participants who indicated that a small amount of beer per day is not harmful, 19.4% drank alcohol twice to four times a month. Second, with regard to beliefs about risks of drinking wine, the 83.3% of women who reported that any wine consumption is harmful indicated no alcohol consumption, while abstinence was reported by half of the participants who said that a small amount of wine per day is not harmful, χ^2^(12, N = 216) = 33.08, *p* = 0.001, γ = −0.43. Among participants who stated that a small amount of wine a day is safe, a third reported alcohol consumption twice to four times a month. No significant relationships with alcohol consumption were observed regarding other variables of risk perception.

### 3.7. Structural Equation Model of Demographic Variables, Risk Perception, and Alcohol Consumption during Pregnancy

In order to determine which of the variables best explain alcohol consumption during pregnancy we developed a structural equation model in which, based on the sociodemographic factors that are most influential on alcohol consumption during pregnancy, such as age, relationship status, and educational level, we sought to determine whether they can modulate the effect of perceived risk of beer and wine consumption over said variable (frequency of consumption). The model sought to explain the frequency of alcohol consumption during pregnancy, assuming the influence of these variables.

Thus, taking into account the previous results, a path analysis was tested in which: (a) risk perception of beer consumption and risk perception of wine consumption were related to the frequency of alcohol consumption during pregnancy; (b) age presented an effect on risk perception of beer consumption while pregnant; (c) being in a relationship was linked to both the risk perception of beer and wine consumption; and (d) educational level presented effects on the risk perception of beer consumption and on the frequency of alcohol consumption during pregnancy. This model did not reach good data fit, Satorra-Bentler χ^2^(8, N = 426) = 197.38, *p* < 0.001, CFI = 0.132, RMSEA = 0.39. Lagrange multipliers test suggested two modifications to improve the fit of the model: (a) an association between age and educational level; and (b) and association between risk perception of beer consumption and risk perception of wine consumption. After these modifications, the model obtained a good overall fit, Satorra-Bentler χ^2^(6, N = 426) = 10.53, *p* = 0.104, CFI = 0.978, RMSEA = 0.06. 

**Figure 6:**
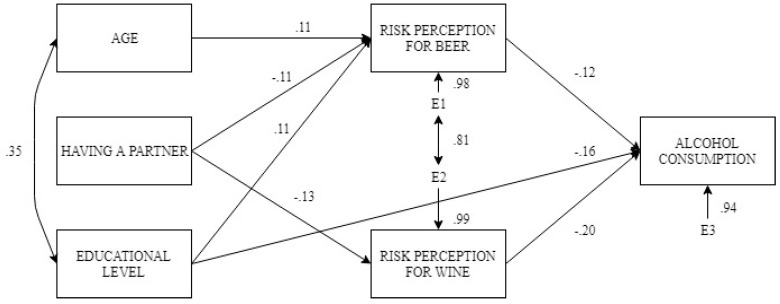
Structural equation model of risk perception and demographics as determinants of frequency of alcohol consumption during pregnancy.

Figure 6 shows the model that reached a good overall fit, indicating the standardised solutions. Thus, a lower risk perception about beer and wine consumption, and a lower educational level, were related to more frequent alcohol consumption. Risk perceptions of beer and wine consumptions were positively associated. Furthermore, being in a relationship was associated with a greater risk perception of drinking beer or wine. Younger participants showed lower risk perception concerning beer consumption. Regarding educational level, higher levels of education were related to a greater risk perception of beer. Finally, the older participants were characterized by having higher educational levels in our sample. In general, the model presented an explained variance of *R*^2^ = 0.122 for the frequency of self-reported alcohol consumption. The risk perception of beer consumption showed an *R*^2^ = 0.043, while the risk perception of wine consumption presented an *R*^2^ = 0.016.

## 4. Discussion

In order to analyze risk perception of alcohol consumption during pregnancy we interviewed a random representative sample of the pregnant women in a community health area of a southern European city (Seville, Spain), who were in their 20th week of pregnancy.

A large portion of the interviewees (90.6%) stated the generic belief that alcohol consumption in pregnancy could be harmful to the baby, or to the baby and to herself. However, it was noted that a quarter of the women were not able to cite any specific risks when asked to do so. Furthermore, only half of them (48%) knew that the teratogenic effects of prenatal exposure to alcohol are life-long. Just over a quarter of the sample (27.5%) indicated that they did not know how long the sequelae of alcohol consumption during pregnancy lasted. Therefore, we can assume that the pregnant women in our sample had, on average, a deficient knowledge regarding the damages that may result from prenatal exposure to alcohol. That probably represents widespread lack of information among women of reproductive age (and among pregnant women in particular) about the teratogenic potential of prenatal alcohol exposure, which in turn could be related to, among other factors, the scarcity of healthcare advice on this topic, either before or during pregnancy, as studies carried out in Australia and Spain suggested [29,33].

This lack of information regarding the harmful effects of alcohol on pregnancy has also been detected in other studies. One example is that conducted by Crawford-Williams in 2015 on various focus groups of pregnant Australian women and their partners. That study concluded that although the majority of the participants knew that alcohol could be harmful during the pregnancy, they had very limited knowledge about the specific damages that it could cause [33]. Furthermore, a telephone survey also conducted in Australia with a representative sample of the women of reproductive age found that although 92.7% of the women surveyed agreed that alcohol consumption during pregnancy could harm the fetus, 16.2% did not agree that said harmful effects could be life-long [23]. On the other hand, a study in Russia on a sample of women of reproductive age showed that 40% of them believed alcohol consumption during pregnancy to be acceptable, or else did not have a clear opinion on the subject. Although one third of them had heard about Fetal Alcohol Syndrome, only 8% of them had any precise knowledge about the condition [34].

In our study, only 70% of the sample indicated that any amount of alcohol consumed during pregnancy is harmful. When questioned about the risks derived from alcohol consumption during pregnancy as a function of the type of drink consumed, they had a low risk perception of fermented beverages (wine and beer) in comparison with distilled beverages, where almost 90% of the women felt that a minimum amount per day is harmful. This may result from a good social image of wine and beer, which would be shared by a large portion of the pregnant women. Similarly, it may be indicative of a reductionist view of the concept of an alcoholic drink, which may be widespread throughout society, which considers that only distilled beverages are truly alcoholic drinks. Additionally, it may also be related to the difficulty that some people have when it comes to handling the concept of alcoholic grade. 

The positive correlation between the education level of pregnant women and risk perception of alcohol consumption in pregnancy has been noted in earlier studies [31]. In our sample, the educational level of pregnant women turned out to be positively associated with risk perception of both distilled beverages and beer. Presumably, it is those women with a university education or with more years of pre-university education who, on the one hand, have more access to quality sources of information, and on the other hand, better retain the information that they receive. In France, in a national survey with a representative sample of the pregnant and early post-partum women of the country, a slight risk perception of the beer and wine consumption during pregnancy was also detected, in contrast with distilled beverages [30]. Moreover, in Australia, a qualitative study of 40 pregnant or puerperal women concluded that in general, the women considered that consuming small amounts of alcohol during pregnancy was a low-risk activity and that, on the other hand, they evaluated the risk as a function of the type of beverage consumed more than as a function of the alcohol content of the beverage. Wine turned out to have a particularly positive image. The study also found that rather than using the concept of a “standard unit of drink”, those women considered the glass of wine as the unit of reference [35]. 

The results from our study suggest that there may be a lower risk perception of the effects of alcohol consumption during pregnancy among multiparous women, compared with first-time mothers. Although the differences found did not reach statistical significance, there is a tendency that suggests that women with previous pregnancies may underestimate the harmful effect of alcohol consumption during pregnancy. A study conducted on pregnant women in the United States who consumed alcohol before learning they were pregnant concluded that the risk perception of alcohol consumption in pregnancy is lower among those women who have previously given birth to a healthy child [26]. The authors interpreted this finding in the sense that, since very often alcohol consumption during pregnancy does not translate into visible abnormalities in the short term, those women who regularly consume alcohol relax during subsequent pregnancies and consider less necessary the precaution of abstaining from alcohol throughout the pregnancy. 

The distribution of risk perception regarding alcohol consumption during pregnancy varies as a function of the pregnant woman’s relationship status. Not being in a relationship is associated with a lower risk perception of the consumption of beer and wine, to the extent that none of the pregnant women in our sample who were not in a relationship said that any beer consumption during pregnancy is harmful, as opposed to one third of the women who were in a relationship. This result proves difficult to interpret. In our sample (randomly selected from among the pregnant women in a community health area), only 2% of the pregnant women were not in a relationship during their 20th week of pregnancy (mostly women in a situation of marital separation). Future studies should delve deeper into this aspect, in order to be able to adequately interpret this association, and determine whether there truly is a need for some type of preventive intervention aimed at single women who are pregnant or about to get pregnant. 

Three quarters of our sample indicated that they had not consumed any alcoholic beverages during the previous 4 or 5 months (a time period which coincides with the length of their pregnancy). The remaining one quarter of the women did indicate that they had consumed alcohol, although in general very sporadically. It is very likely that among the interviewees alcohol consumption would have been under reported, for reasons of social desirability, or forgetting or not being aware that fermented drinks are also alcoholic beverages. Those studies that evaluated prenatal alcohol exposure using biomarkers as well as questionnaires have shown a tendency toward underreporting when the data refer to self-reported alcohol consumption, since the biomarkers detect a notably higher prevalence [36]. In a recent study of 153 pregnant women who received care at a university hospital in Barcelona (Spain) the analysis of hair samples concluded that only 35% of the women had been fully abstinent during their pregnancy [37]. However, there are studies based on questionnaires that have detected significantly high rates of consumption. In particular, a cohort study conducted in Australia of a random sample of 1969 pregnant women throughout the entire country found that 82% of the women surveyed reported having continued drinking during the pregnancy [38].

On the other hand, in our study, the educational level of the pregnant women was inversely correlated with the self-reported frequency of alcohol consumption. In contrast, a 2002 study conducted in New Zealand found that women from a lower socio-economic level were the ones who most often reported having consumed alcohol during their pregnancy [39]. These discrepancies are difficult to interpret. They may reflect differences between countries in terms of the patterns of alcohol consumption among women of reproductive age, or else a greater awareness of the risk among women with a higher educational level, or even a greater tendency to bias their responses as a function of social desirability. The latter possibility could be clarified in future studies through the evaluation of alcohol consumption using biomarkers. 

One finding in our study that is particularly relevant for prevention is that risk perception of alcohol consumption during pregnancy is negatively correlated with self-reported frequency of alcohol consumption during pregnancy. This was especially noted in the case of wine and beer, maybe because the risk perception of consuming fermented beverages during pregnancy varies more than the risk perception concerning the consumption of distilled beverages. A study by Testa and Reifman (1996) [26] also found an inverse association between risk perception during pregnancy and alcohol consumption during the current pregnancy. 

Receiving quality information (true, precise, and understandable) about the risks derived from consuming alcoholic beverages during pregnancy (from the very beginning of the pregnancy) is a right of any woman of childbearing age, as it is a crucially important measure for the prevention of FASD. In this sense, our study points out that a very superficial or even erroneous view of the risks derived from alcohol consumption during pregnancy is prevalent among pregnant women, and that there is, in turn, an inverse relationship between this risk perception and the self-reported frequency of alcohol consumption during pregnancy. A crucial step to modify this situation is that the country’s official health guidelines clearly recommend, to both pregnant women and women who are about to get pregnant, the need to avoid any type of alcohol consumption during pregnancy, as is already being done in an increasing number of countries [40,41]. Similarly, it is necessary to formulate plans for the continued education of all the health professionals who provide pre-pregnancy or pregnancy care, so that they may feel motivated and prepared to properly evaluate alcohol consumption among pregnant women and women about to get pregnant and can provide timely education on the matter [28,42,43]. Healthcare institutions should establish protocols that standardize these practices and articulate programs that facilitate their correct implementation on the part of healthcare personnel, particularly when providing care for women with low educational levels who, as our study has shown, are the ones that are least aware of the risk inherent in drinking alcoholic beverages while pregnant. 

Even if practically all health professionals make the most out of the opportunities to prevent FASD that are offered in everyday clinical practice, it is not realistic to think that a vast majority of pregnant women, or women who are about to get pregnant, are going to abstain from drinking alcoholic beverages. The best predictor of alcohol consumption during pregnancy is the consumption prior to the pregnancy [31]. This finding, common to a variety of studies, suggests that there is a trend to continuity in this aspect of lifestyle between pre-conception and gestation—something not surprising taking into account that alcohol may produce dependence and that alcohol intake is a practice socially promoted in many countries. From this perspective, the prevention of prenatal exposure to alcohol should begin before pregnancy, applying proven-effective measures for reducing per capita alcohol consumption, especially those measures with higher impact on young people’s consumption [42,43,44,45,46,47,48,49,50,51]. Within a social context where drinking alcohol proves to be less affordable, less physically accessible, and less socially prestigious, per capita consumption of alcohol among people in childbearing age (including women) will be lower and, therefore, the presence of this strong predictor of prenatal alcohol exposure will be weaker. If, in addition, choosing not to drink alcohol during pregnancy or while planning a pregnancy is socially praised, the prevention of a significant portion of future children suffering from prenatal exposure to alcohol would prove more feasible.

In light of the results of this and other studies [30], it is apparent that there is a need to spread the message in society that fermented drinks (wine, beer, cider, etc.) are also alcoholic beverages and that their consumption may entail multiple risks (not just with respect to pregnancy, but also in many other aspects such as the risk for breast cancer, particularly when they are consumed on a regular basis between menarche and the first pregnancy [50,51]).

This study has both strengths and limitations. The strengths include a randomly selected sample of participants from among the pregnant women who were receiving care at the outpatient clinics of a university hospital, and that the data collection was done through face-to-face interviews conducted by healthcare personnel trained specifically for that purpose. However, given that this is a cross-sectional study, it is not possible to infer any causal relationships between the variables. In addition, although the sample was randomly chosen, only 51.2% of the pregnant women selected agreed to participate. Migrant women who were not fluid in Spanish were excluded from the sample due to lack of available interpreting services. Alcohol consumption was evaluated only through self-reports, not also through the analysis of metabolites in biological samples. The relationship between risk perception and the type/amount of information given by health care providers was not explored. Moreover, some response categories related to risk perception of alcohol consumption during pregnancy could be biased by a subjective interpretation of the quantity i.e., when answering “Drinking a small amount every day is not harmful”, or when they conflated the amount of alcohol consumed (not specified in terms of ml or ounces) and the frequency of the drinking episodes. Nevertheless, it should be remembered that in Mediterranean countries such as Spain there is no historic tradition of serving exact volumes of alcohol beverages in bars. This fact could significantly reduce the feasibility of formulating questions, in the context of a brief interview, regarding risk perception of alcohol consumption taking into account exact volume measures.

## 5. Conclusions

Among the conclusions derived from the study, we were able to establish that pregnant women were less aware of the risks involving the potential teratogenic effects from consuming beer and wine during pregnancy, compared to the risks of consuming distilled beverages. On the other hand, risk perception of alcohol consumption during pregnancy was positively correlated with educational level. In turn, risk perception was inversely related with the self-reported frequency of alcohol consumption during pregnancy. Similarly, at least in terms of this sample of pregnant women from southern Europe, the lower the educational level, the greater the self-reported consumption of alcohol during pregnancy.

## Figures and Tables

**Table 1:** Descriptive characteristics of the sample and frequency of alcohol consumption.

Variables	Categories	Percentage
**Age**	Up to 25 years old	12.4
	26–30 years old	23.4
	31–35 years old	38.0
	More than 35 years old	26.3
**Educational level**	Low level of studies	16.9
	Medium level of studies	45.5
	University studies	37.6
**Employment status**	Full time employment	39.3
	Part time employment	12.7
	Unemployed	28.0
	Other employment status	20.0
**In a relationship**	Yes	98.1
	No	1.9
**Number of pregnancies (including this one)**	One	40.4
	Two	31.0
	More than two	28.6
**Was pregnancy planned?**	Yes	74.6
	No	25.4
**Self-reported frequency of alcohol consumption during pregnancy**	Never	75.4
	Once a month or less	14.6
	Between 2–4 times a month	8.4
	Between 2–3 times a week	1.2
	Nonspecific response	0.5

**Table 2:** Risk perception of alcohol consumption during pregnancy.

Variables	Categories	Percentage
Risk perception of alcohol consumption to the baby, mother.	Yes, to the baby	48
	Yes, to myself (mother)	0.2
	Yes, to both	42.6
	Yes, if I abuse it	3.8
	I don t know	1.5
	No, if it s moderated	2.8
	Nonspecific response	0.9
Risk of malformations, miscarriage or abortion	Yes	72.9
Risk of premature delivery	Yes	2.5
Risk of abstinence syndrome	Yes	12.9
Risk for problems in the baby’s development	Yes	24.6
Duration of the complications affecting the baby	Only throughout the pregnancy	2.4
	Only at birth	3.8
	Up to the early years	9.3
	For many years	5.2
	Entire life	48.1
	Does not know	27.5
	Other responses	3.8
Risk perception of consuming alcoholic drinks (in general) during pregnancy	Any amount is harmful	69.7
	<Once/month is not harmful	16.9
	<Once a week is not harmful	9.1
	A small amount/day is not harmful	3.9
		0.4
Risk perception of drinking beer during pregnancy	Any amount is harmful	31.5
	<Once/month is not harmful	27.6
	<Once a week is not harmful	25.3
	A small amount/day is not harmful	14.8
	Any amount is not harmful	0.8
Risk perception of drinking wine during pregnancy	Any amount is harmful	38.4
	<Once/month is not harmful	30.6
	<Once a week is not harmful	24.2
	A small amount/day is not harmful	5.9
	Any amount is not harmful	0.9
Risk perception of drinking spirits during pregnancy	Any amount is harmful	88
	<Once/month is not harmful	5.7
	<Once a week is not harmful	4.4
	A small amount/day is not harmful	1.9

## References

[1] Lemoine P., Harousseau H., Borteyru J.P., Menuet J.C. (1968). Les enfants des parents alcooliques: Anomalies observes a propos de 127 cas. Ouest-Médical..

[2] Jones K., Smith D. (1973). Recognition of the fetal alcohol syndrome in early infancy. Lancet.

[3] Dörrie N., Föcker M., Freunscht I., Hebebrand J. (2014). Fetal alcohol spectrum disorders. Eur. Child Adolesc. Psychiatry.

[4] Hoyme H., Kalberg W., Elliott A., Blankenship J., Buckley D., Marais A., Manning M.A., Robinson L.K., Adam M.P., Abdul-Rahman O. (2016). Updated Clinical Guidelines for Diagnosing Fetal Alcohol Spectrum Disorders. Pediatrics.

[5] Williams J., Smith V. (2015). Fetal Alcohol Spectrum Disorders. Pediatrics.

[6] Denny L., Coles S., Blitz R. (2017). Fetal Alcohol Syndrome and Fetal Alcohol Spectrum Disorders. Am. Fam. Physician.

[7] Bailey B.A., Sokol R. (2011). Prenatal alcohol exposure and miscarriage, stillbirth, preterm delivery, and sudden infant death syndrome. Alcohol Res. Health.

[8] Cook J., Green C., Lilley C., Anderson S., Baldwin M., Chudley A., Conry J., LeBlanc N., Loock J., Lutke J. (2015). Fetal alcohol spectrum disorder: A guideline for diagnosis across the lifespan. Can. Med. Assoc. J..

[9] Mamluk L., Edwards H., Savović J., Leach V., Jones T., Moore T., Sharea I., Sarah J.L., Jenny L.D., Debbie L. (2017). Low alcohol consumption and pregnancy and childhood outcomes: Time to change guidelines indicating apparently ‘safe’ levels of alcohol during pregnancy? A systematic review and meta-analyses. BMJ Open.

[10] Sokol R. (1981). Alcohol and abnormal outcome of pregnancy. Can. med. assoc. J..

[11] Sokol R. (2003). Fetal Alcohol Spectrum Disorder. J. Am. Med. Assoc..

[12] Streissguth A., Landesman-dwyer S., Martin J., Smith D. (1981). Teratogenic Effects of Alcohol in Humans and Laboratory Animals. Obstet. Gynecol. Surv..

[13] Streissguth A.P., Bookstein F.L., Barr H.M., Sampson P.D., O’Malley K., Young J.K. (2004). Risk factors for adverse life outcomes in fetal alcohol syndrome and fetal alcohol effects. J. Dev. Behav. Pediatr..

[14] Conover E., Jones K. (2012). Safety concerns regarding binge drinking in pregnancy: A review. Birth Defects Res. A. Clin. Mol. Teratol..

[15] Deshpande S., Basil M., Basford L., Thorpe K., Piquette-Tomei N., Droessler J., Cardwell K., Williams R.J., Bureau A. (2005). Promoting Alcohol Abstinence Among Pregnant Women. Health Marketing Quart..

[16] Henderson J., Kesmodel U., Gray R. (2007). Systematic review of the fetal effects of prenatal binge-drinking. J. Epidemiol. Community Health.

[17] Vall O., Salat-Batlle J., Garcia-Algar O. (2015). Alcohol consumption during pregnancy and adverse neurodevelopmental outcomes. J. Epidemiol. Community Health.

[18] Popova S., Lange S., Probst C., Gmel G., Rehm J. (2017). Estimation of national, regional, and global prevalence of alcohol use during pregnancy and fetal alcohol syndrome: A systematic review and meta-analysis. Lancet. Glob. Health.

[19] Howlett H., Abernethy S., Brown N., Rankin J., Gray W. (2017). How strong is the evidence for using blood biomarkers alone to screen for alcohol consumption during pregnancy? A systematic review. Eur. J. Obstet. Gynecol. Reprod. Biol..

[20] Howlett H., Mackenzie S., Gray W., Rankin J., Nixon L., Richardson A., Strehle E., Browna N.W. (2018). Assessing prevalence of alcohol consumption in early pregnancy: Self-report compared to blood biomarker analysis. Eur. J. Med. Genet.

[21] Cordovilla-Guardia S., Guerrero-López F., Maldonado A., Vilar-López R., Salmerón J., Romero I., Pose S., Fernández-Modéjar E. (2014). Trauma risk perception related to alcohol, cannabis, and cocaine intake. Eur. J. Trauma Emerg. Surg..

[22] Galván G., Sánchez-Carballo Á., Gómez-Morales I., Humánez-Julio O., Guerrero-Martelo M., Vásquez De la Hoz F. (2016). Belief system regarding Cannabis, its use and consequences: Users versus non-users in colombian university students. Vertex.

[23] Oshi S.N., Abel W.D., Ricketts Roomes T., Meka I.A., Harrison J., Weaver S., Agu C.F., Smith P.W., Omeje J.C., Rae T. (2018). Does Risk Perception Affect Alcohol Consumption among Secondary School Students in Jamaica?. Asian Pac. J. Cancer Prev..

[24] Valente P., Mipatrini D., Mannocci A., Ruscitti L.E., Sernia S., Ceccanti M., La Torre G. (2018). Perception of alcohol problem among workers of the transportation, healthcare and building sectors in the Lazio Region. Med. Lav..

[25] Peadon E., Payne J., Henley N., D’Antoine H., Bartu A., O’Leary C., Bower C., Elliott E.J. (2010). Women’s knowledge and attitudes regarding alcohol consumption in pregnancy: A national survey. BMC Public Health.

[26] Testa M., Reifman A. (1996). Individual differences in perceived riskiness of drinking in pregnancy: Antecedents and consequences. J. Stud. Alcohol.

[27] O’Connor M., Whaley S. (2007). Brief Intervention for Alcohol Use by Pregnant Women. Am. J. Public Health.

[28] Anderson A., Hure A., Kay-Lambkin F., Loxton D. (2014). Women’s perceptions of information about alcohol use during pregnancy: A qualitative study. BMC Public Health.

[29] Mendoza R., Morales-Marente E., Palacios M., Rodríguez-Reinado C., Corrales-Gutiérrez I., García-Algar Ó. (2019). Health advice on alcohol consumption in pregnant women in Seville (Spain). Gaceta Sanitaria.

[30] Dumas A., Toutain S., Hill C., Simmat-Durand L. (2018). Warning about drinking during pregnancy: Lessons from the French experience. Reproductive Health.

[31] Skagerstróm J., Chang G., Nilsen P. (2011). Predictors of Drinking During Pregnancy: A Systematic Review. J. Womens Health.

[32] Babor T.F., Higgins-Biddle J.C., Saunders J.B., Monteiro M.G. (2001). The Alcohol Use Disorders. Identification Test. Guidelines for Use in Primary Care.

[33] Crawford-Williams F., Steen M., Esterman A., Fielder A., Mikocka-Walus A. (2015). “My midwife said that having a glass of red wine was actually better for the baby”: A focus group study of women and their partner’s knowledge and experiences relating to alcohol consumption in pregnancy. BMC Pregnancy Childbirth.

[34] Balachova T., Bard D., Bonner B., Chaffin M., Isurina G., Tsvetkova L., Volkova E. (2016). Do attitudes and knowledge predict at-risk drinking among Russian women?. Am. J. Drug Alcohol Abuse.

[35] Meurk C., Broom A., Adams J., Hall W., Lucke J. (2014). Factors influencing women’s decisions to drink alcohol during pregnancy: Findings of a qualitative study with implications for health communication. BMC Pregnancy Childbirth..

[36] Chiandetti A., Hernandez G., Mercadal-Hally M., Alvarez A., Andreu-Fernandez V., Navarro-Tapia E., Bastons-Compta A., Garcia-Algar O. (2017). Prevalence of prenatal exposure to substances of abuse: Questionnaire versus biomarkers. Reproductive Health..

[37] Gomez-Roig M., Marchei E., Sabra S., Busardò F., Mastrobattista L., Pichini S., Gratacós E., Garcia-Algar O. (2018). Maternal hair testing to disclose self-misreporting in drinking and smoking behavior during pregnancy. Alcohol..

[38] Anderson A., Hure A., Forder P., Powers J., Kay-Lambkin F., Loxton D. (2013). Predictors of antenatal alcohol use among Australian women: A prospective cohort study. BJOG.

[39] McLeod D., Pullon S., Cookson T., Cornford E. (2002). Factors influencing alcohol consumption during pregnancy and after giving birth. N. Z. Med. J..

[40] National Health and Medical Research Council (2009). Australian Guidelines to Reduce Health Risks from Drinking Alcohol.

[41] Kesmodel U., Kesmodel P. (2011). Alcohol in Pregnancy: Attitudes, Knowledge, and Information Practice Among Midwives in Denmark 2000 to 2009. Alcohol Clin. Exp. Res..

[42] Payne J., Elliott E., D’Antoine H., O’Leary C., Mahony A., Haan E., Bower C. (2005). Health professionals’ knowledge, practice and opinions about fetal alcohol syndrome and alcohol consumption in pregnancy. Aust. N. Z. J. Public Health.

[43] Payne J., Watkins R., Jones H., Reibel T., Mutch R., Wilkins A., Whitlock J., Bower C. (2014). Midwives’ knowledge, attitudes and practice about alcohol exposure and the risk of fetal alcohol spectrum disorder. BMC Pregnancy and Childbirth.

[44] Anderson P., Chisholm D., Fuhr D.C. (2009). Effectiveness and cost-effectiveness of policies and programmes to reduce the harm caused by alcohol. Lancet.

[45] Elder R., Lawrence B., Ferguson A., Naimi T., Brewer R., Chattopadhyay S., Toomey T.L., Fielding J.E. (2010). The Effectiveness of Tax Policy Interventions for Reducing Excessive Alcohol Consumption and Related Harms. Am. J. Prev. Med..

[46] Hahn R.A., Kuzara J.L., Elder R., Brewer R., Chattopadhyay S., Fielding J., Naimi T.S., Toomey T., Middleton J.C., Lawrence B. (2010). Effectiveness of policies restricting hours of alcohol sales in preventing excessive alcohol consumption and related harms. Am. J. Prev. Med..

[47] Middleton J.C., Hahn R.A., Kuzara J.L., Elder R., Brewer R., Chattopadhyay S., Fielding J., Naimi T.S., Toomey T., Lawrence B. (2010). Effectiveness of policies maintaining or restricting days of alcohol sales on excessive alcohol consumption and related harms. Am. J. Prev. Med..

[48] Wagenaar A., Salois M., Komro K. (2009). Effects of beverage alcohol price and tax levels on drinking: A meta-analysis of 1003 estimates from 112 studies. Addiction.

[49] Xuan Z., Chaloupka F.J., Blanchette J.G., Nguyen T.H., Heeren T.C., Nelson T.F., Naimi T.S. (2015). The relationship between alcohol taxes and binge drinking: Evaluating new tax measures incorporating multiple tax and beverage types. Addiction.

[50] Liu Y., Colditz G.A., Rosner B., Berkey C.S., Collins L.C., Schnitt S.J., Connolly J.L., Chen W.Y., Willett W.C., Tamimi R.M. (2013). Alcohol intake between menarche and first pregnancy: A prospective study of breast cancer risk. J. Natl. Cancer Inst..

[51] Liu Y., Nguyen N., Colditz G.A. (2015). Links between alcohol consumption and breast cancer: A look at the evidence. Womens Health.

